# Comparative Wear Evaluation of Pure Zn, Zn–Mg and Zn–Mg–Y Alloys Using Mass Loss Measurements and Optical Profilometry

**DOI:** 10.3390/ma19061211

**Published:** 2026-03-19

**Authors:** Traian-Lucian Severin, Viorel Paleu, Costică Bejinariu, Catrinel-Raluca Giurma-Handley, Ioan Tamasag, Nicanor Cimpoesu, Stefan Constantin Lupescu, Georgeta Zegan, Ana-Maria Roman, Gheorghe Bădărău, Nicoleta Ioanid

**Affiliations:** 1 Faculty of Mechanical Engineering, Automotive and Robotics, “Ștefan cel Mare” University of Suceava, 720229 Suceava, Romania; severin.traian@usm.ro (T.-L.S.); ioan.tamasag@usm.ro (I.T.); stefan.lupescu@usv.ro (S.C.L.); 2 Faculty of Mechanical Engineering, “Gheorghe Asachi” Technical University of Iasi, 43 Dimitrie Mangeron Blvd., 700050 Iasi, Romania; viorel.paleu@academic.tuiasi.ro; 3 Faculty of Materials Science and Engineering, “Gheorghe Asachi” Technical University of Iasi, 41 Dimitrie Mangeron Blvd., 700050 Iasi, Romania; nicanor.cimpoesu@academic.tuiasi.ro (N.C.); ana-maria.roman@academic.tuiasi.ro (A.-M.R.); gheorghe.badarau@academic.tuiasi.ro (G.B.); 4 Academy of Romanian Scientists, Ilfov 3, 050044 Bucharest, Romania; 5 Faculty of Hydrotechnical Engineering, Geodesy and Environmental Engineering, Gheorghe Asachi Technical University of Iasi, 63–65 Prof. Dr. Doc. Dimitrie Mangeron Blvd., 700050 Iasi, Romania; catrinel-raluca.giurma-handley@academic.tuiasi.ro; 6 Faculty of Dental Medicine, Grigore T. Popa University of Medicine and Pharmacy Iasi, 700115 Iasi, Romania; nicoleta.ioanid@umfiasi.ro

**Keywords:** biodegradable Zn alloys, wear resistance, tribological testing, friction

## Abstract

The present study investigates the dry sliding wear behaviour of pure Zn, Zn–3Mg, and Zn–3Mg–0.5Y biodegradable alloys using mass loss measurements, friction torque monitoring on an Amsler tribometer, and optical profilometry of wear tracks. The microstructure of the Zn–Mg–Y alloy exhibited an α-Zn matrix comprising Zn–Mg intermetallic constituents and dispersed Y-rich phases. Tribological testing at 20 N and 30 N revealed a marked enhancement in wear resistance for Zn–3Mg in comparison to pure Zn, attributable to matrix strengthening by intermetallic phases. Despite the stabilising effect of Y on the friction response, there was no consistent reduction in wear volume under higher loads. Surface investigations have revealed a multifaceted wear mechanism, characterised by a combination of abrasion, oxide tribolayer formation, and localised adhesion. The measured wear rates were found to fall within the range documented in the available literature concerning biodegradable Zn-based alloys, thereby confirming the experimental validity of the findings. In summary, Zn–3Mg exhibited the optimal equilibrium between friction stability and wear resistance under the examined dry sliding conditions. However, further research in physiological environments is necessary to evaluate its biomedical applicability.

## 1. Introduction

Current medical practice employs implants primarily manufactured from permanent metallic materials (stainless steel, titanium alloys, Co–Cr alloys) or non-resorbable non-metallic materials and bioresorbable polymers. However, a significant proportion of these can now be replaced or functionally supplemented by biodegradable metal alloys based on magnesium, zinc, or iron [1,2]. These implants primarily consist of temporary osteosynthesis devices, including screws, plates, pins, broaches, staples, suture anchors, and interference screws, used in orthopaedics, traumatology, maxillofacial surgery, and sports orthopaedics [3,4,5]. The primary function of these materials is to provide adequate mechanical stability during the initial phase of bone or tissue healing. Subsequent to this, the material undergoes progressive degradation and resorption, thereby obviating the necessity for surgical removal [6]. The fastening systems employed vary depending on the application, location, and purpose of implantation. Such systems include plates and screws for rigid or relatively elastic stabilization of fractures, rods or pins for alignment and temporary support, interference screws for fixing ligament grafts, and threaded or press-fit anchors for reattaching soft tissue to bone [7,8]. Magnesium alloys are the preferred option for applications with moderate mechanical demands and relatively short support duration, while zinc alloys offer more controlled degradation and good biocompatibility. Iron alloys are being investigated for situations requiring longer-term mechanical support [9]. Overall, it can be concluded that the utilisation of biodegradable alloys is consistent with a contemporary and rational approach to implantology, which is centred on temporary functionality, biological compatibility, and the mitigation of long-term complications associated with permanent implants [10].

The phenomenon of implant wear is particularly important in assessing the performance of implantable materials. Factors such as the implantation site, the type of local biomechanical stresses, and the fixation system used are all closely dependent on this process [11,12]. Implants placed in areas of repetitive movement and complex cyclic loads, such as large joints (hip, knee, ankle) or bone–soft tissue transition areas, are exposed to wear mechanisms through friction, fatigue, micro-sliding and fretting corrosion. In contrast, implants intended for rigid osteosynthesis, which are fixed stably by plates and screws, are less affected by classic wear, but may suffer local degradation at the implant–bone or implant–screw interface [13].

It is acknowledged that fixation systems which facilitate a certain degree of micro-movement, including suture anchors, interference screws and semi-rigid constructs, have been observed to promote fretting wear. This process involves the release of metal particles or degradation products, a phenomenon with the potential to impact the local biological response [14]. When considering biodegradable alloys, mechanical wear is frequently associated with electrochemical corrosion processes. In order to comprehensively assess this phenomenon, it is essential to integrate a detailed analysis of tribological behaviour and mechanical stability within the physiological environment [15]. Consequently, conducting wear resistance tests that are adapted to real implantation conditions (i.e., cyclic loading, a biological simulation environment, specific geometries and fixation systems) is imperative for validating the safety and durability of implantable alloys. The objective is to ensure an optimal balance between initial strength, degradation rate and biological compatibility throughout the healing process [16].

Despite the inevitable exposure of biodegradable metallic implants to physiological fluids, dry sliding conditions maintain relevance for specific and clinically realistic contact scenarios. During the implantation process, initial fixation, and the early postoperative stages, there is a possibility of localized metal-to-metal or metal-to-bone contacts occurring under conditions of limited lubrication. This is particularly notable at screw–plate interfaces, implant–implant junctions, threaded regions, and micro-contact zones that are susceptible to fretting or micromotion. In such situations, fluid alone is often insufficient to ensure full lubrication, and contact is governed by asperity interaction, oxide film formation, and third-body debris, which are effectively captured under dry sliding conditions [17]. Furthermore, dry wear testing is a conservative and reproducible approach for isolating the intrinsic tribological response of the material and the influence of microstructure, without the additional variables introduced by corrosion processes. Consequently, dry sliding tests are widely utilised as an initial screening step for biodegradable alloys, providing fundamental insight into friction stability, wear mechanisms and load sensitivity. These are essential for understanding early-stage implant performance and for designing subsequent combined wear–corrosion investigations under simulated physiological environments [18,19].

The present study investigates the wear resistance of Zn, ZnMg and ZnMgY materials in a dry regime, at varying forces. From a medical perspective, few of these scenarios have been the subject of discussion.

## 2. Materials and Methods

Samples were obtained through an experimental process involving the melting of pure Zn (99.995%) and master-alloys ZnMg and MgY master-alloys in an electric furnace. The complete process for obtaining the alloy is detailed in a previous study in reference [20].

The Zn3Mg0.5Y alloy was hot rolled in its cast condition with low reduction rates below 10% and at a rolling speed of 30 rpm. The sample underwent 10 passes with different reduction rates at a heating temperature of 250 °C for 10 min, resulting in a final thickness of 1 mm. The first two passes used a higher reduction rate of approximately 10%, followed by lower reduction rates of around 5%. Samples processed by rolling had a thickness of 4 mm, which represented a reduction of approximately 60% from 10 mm.

A series of parallelepiped samples measuring 50 mm × 10 mm × 3 mm were obtained by cutting the base material of pure Zn, ZnMg alloy in cast state and Zn3Mg0.5Y in cast and hot-rolled state. The tribological performance of the stationary parallelepiped specimens was tested by placing them at the upper point of the tribological contact of the machine [21]. The AMSLER machine’s lower disc (AMSLER & Co., Schaffhouse, Switzerland), which rotates, is manufactured from AISI 52100 bearing steel. This steel has a hardness of 62–65 HRc and has a diameter of 59 mm both radially and axially.

**Figure 1:**
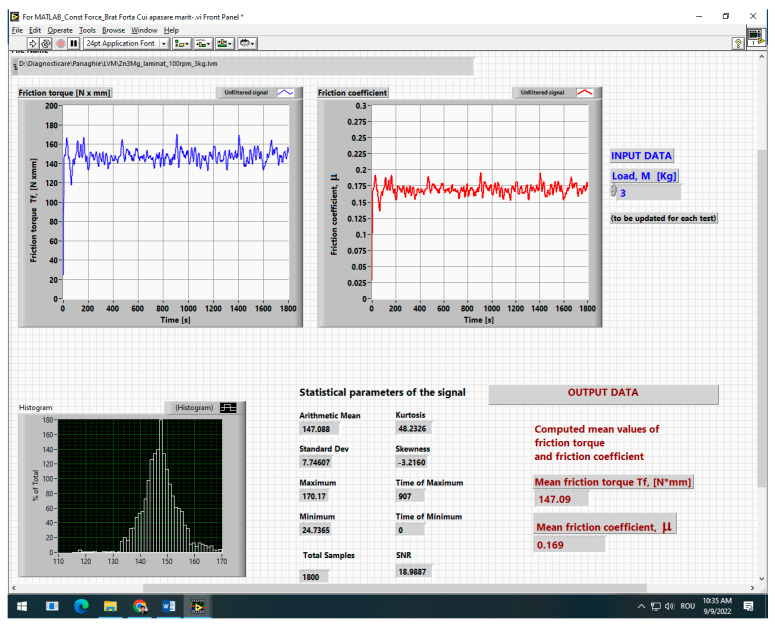
Image of the LabVIEW interface used to collect data on the AMSLER machine (dry lubrication, sample Zn3Mg hot-rolled, speed 100 rpm, load 30 N).

The AMSLER tribometer possesses the capacity to evaluate a wide range of sample geometries, encompassing disc-on-disc, shoe-on-disc, and pad-on-disc configurations. The parameter that is measured is the friction torque. The application of the load was facilitated by the utilisation of dead weights. The measurement of friction torque has been accomplished through the utilisation of tensometric measurements in conjunction with a Vishay P3 strain gage bridge (manufactured by Vishay, Selb, Germany). The implementation of the sixth-order Savitzky–Golay filter within the LabVIEW interface to facilitate the post-processing of data resulted in the outcomes depicted in Figure 1. The design and construction of the AMSLER tribometer are described in detail in [21]. The friction torque signal was post-processed using a sixth-order Savitzky–Golay filter implemented in the LabVIEW interface. A fixed window length was applied for all tests in order to ensure consistent signal smoothing while preserving the characteristic trends of the MoF(t) curves. The filtering procedure was used only to reduce high-frequency noise and did not modify the average friction values. The mean friction coefficient and friction torque reported in this study were therefore calculated from the stabilized steady-state region of the signal, and comparisons between materials were verified to be consistent for both raw and filtered data.

The input data comprise the applied load, a value adjusted for each specific test, and the diameter of the rotating lower disc, which remains constant. The upper specimen was a stationary Zn-based alloy sample (pad configuration). The resulting output data comprise the mean friction torque, mean friction coefficient, and the statistical parameters of the acquired signal. Each test was conducted for 30 min. Each test was conducted at a constant speed of N = 100 rpm for the lower disc. The applied load for the dry tests was 20 N and 30 N, respectively. For each tribological test, the recorded friction torque signal was characterized by an initial running-in stage followed by a more stable regime. The steady-state friction response was evaluated by excluding the initial transient part of the MoF(t) curve and averaging the stabilized region of the signal. In the present work, the reported friction behaviour is therefore based on the steady-state segment after run-in exclusion, while the mass loss and wear rate values correspond to one tested specimen for each condition.

When the signal-to-noise ratio (SNR) exceeds 2, it is indicative of optimal acquisition quality for the friction torque signal. The acquisition quality is considered satisfactory when the SNR is approximately 1. For Gaussian signals, the kurtosis is 3; a K value greater than 3 indicates well-centred data with low noise. The mass of the samples before and after the wear tests was measured using an analytical balance with a precision of ±0.001 g in order to determine the mass loss during the tribological experiments.

**Figure 2:**
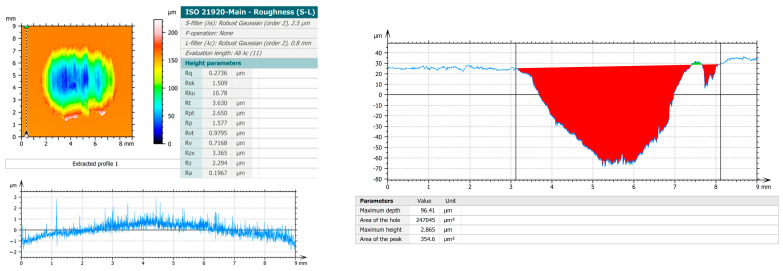
Experimental set-up for profile investigation.

Scanning electron microscopy (SEM) was utilised in this study, employing the VegaTescan LMH II instrument, equipped with a secondary electron (SE) detector and operating at 30 kV with a working distance (WD) of 15.5 mm. Energy-dispersive X-ray spectroscopy (EDS) analysis (automatic and element list modes) was performed using a Bruker XFlash detector attached to the SEM (VegaTescan LMH II) in order to determine the local chemical composition (Point and Mapping analysis approaches) of the investigated microstructural regions. Several measurements were carried out in different areas of the sample to obtain representative average values of the main alloying elements. It should be noted that the purpose of the EDS analysis in the present study was to support the microstructural interpretation by identifying the elemental distribution, while the global chemical composition and structural characteristics of Zn_3_Mg–xY alloys produced by the same processing route were previously investigated and reported in detail in Ref. [20]. The evaluation of surface roughness was conducted utilising a non-contact optical profilometer (Nanovea PS50, Nanovea, Irvine, CA, USA), Figure 2, equipped with dedicated control software (Nanovea 3D, v. 5.7.8.31265). The measurement principle is based on chromatic confocal optical technology, where surface height is determined from the wavelength of reflected white light focused at different vertical positions. The scanning of each deformed surface was conducted individually using a chromatic confocal optical sensor, with a max height range of 300 µm, a working distance of 10.8 mm, and a lateral X–Y accuracy of 1.7 µm. To achieve high measurement precision and to ensure complete coverage of the deformed area, scans were performed with a step size of 2 µm in both the X and Y directions over a 9 × 9 mm surface area. The topography data that had been acquired was processed using specialized surface analysis software (Professional 3D V10, Digital Surf, Besançon, France). The surface texture parameters were determined in accordance with ISO 25178-2 [22], employing a 2.5 µm Gaussian S-filter and a 0.8 mm robust Gaussian L-filter. All experiments were conducted in accordance with the prevailing occupational health and safety laws and regulations, with the objective of eliminating all potential risks and dangers that could have affected human resources during the experimental procedures [23].

While previous studies by the authors focused on the general corrosion mechanisms and basic mechanical properties of the Zn-Mg-Y system, the present work provides a detailed, novel analysis of the long-term tribological performance under variable load conditions of 20 N and 30 N. Specifically, this study highlights the transition from oxidative to adhesive wear, a phenomenon not previously quantified for these specific compositions [20,21,24].

## 3. Results and Discussion

The tribological results presented in this section should be interpreted primarily as a comparative evaluation of the intrinsic wear behaviour of the investigated alloys under controlled dry sliding conditions. These experiments are intended as an initial screening step and do not reproduce the full complexity of physiological environments, where corrosion processes, biological lubrication and cyclic loading may significantly modify wear mechanisms.

The EDS results (Table 1) revealed the chemical composition of the alloy (≈96.8 wt% Zn; ≈2.6 wt% Mg; ≈0.6 wt% Y, with local variations), which was obtained through five determinations from different areas of 1 mm^2^. The spectrum confirmed the dominant presence of Zn (K peaks), with lower contributions of Mg (low K/L peaks) and Y (L series in the ~2 keV area and K series around ~15–17 keV). This suggests that the Zn–Mg–Y alloy can be structurally described as a strongly Zn-rich alloy with an α-Zn (hcp) matrix and a secondary fraction of intermetallic phases formed from alloying elements in excess of their solubility in Zn. Based on the chemical composition measured by EDS after casting (≈96–97 wt.% Zn, ≈2–3 wt.% Mg and ≈0.5 wt.% Y), the phase constitution of the Zn3Mg0.5Y alloy can be interpreted according to the ZnMg and ZnY phase diagrams reported in the literature. The microstructure is expected to consist predominantly of an α-Zn matrix, together with Zn–Mg intermetallic phases formed due to the limited solubility of Mg in Zn near the solidification temperature, most commonly Mg_2_Zn_11_ and/or MgZn_2_. In addition, the presence of Y may lead to the formation of ZnY intermetallic particles such as Zn_3_Y or Zn_12_Y, generally located at interdendritic regions or grain boundaries. Therefore, the as-cast alloy can be described as an α-Zn matrix containing interdendritic ZnMg intermetallics and dispersed Y-rich phases.

As indicated by Zn–Mg binary diagrams, the solubility of Mg in α-Zn is limited at temperatures proximate to solidification. Consequently, at Mg contents of ~2–3 wt%, it is anticipated that, upon cooling, Mg will segregate into the interdendritic zones, resulting in the formation of eutectic Zn + intermetallic Zn–Mg constituents, namely Mg_2_Zn_11_ and/or MgZn_2_ [24]. This is contingent on the thermal history, cooling rate, and local equilibrium, with the potential for the presence of fine precipitates in the vicinity of grain boundaries.

The presence of Y, resulting from the MgY master-alloy, has been shown to promote the formation of stable Zn–Y phases at low Y contents. These phases typically manifest as dispersed particles or accumulations at grain boundaries (e.g., Zn3Y, Zn12Y [24], or other closely related Zn–Y intermetallic compounds). This phenomenon can be attributed to the increased affinity of Y for Zn, which results in the attraction of Y atoms into compounds with a higher melting point and reduced diffusion rate.

**Figure 3:**
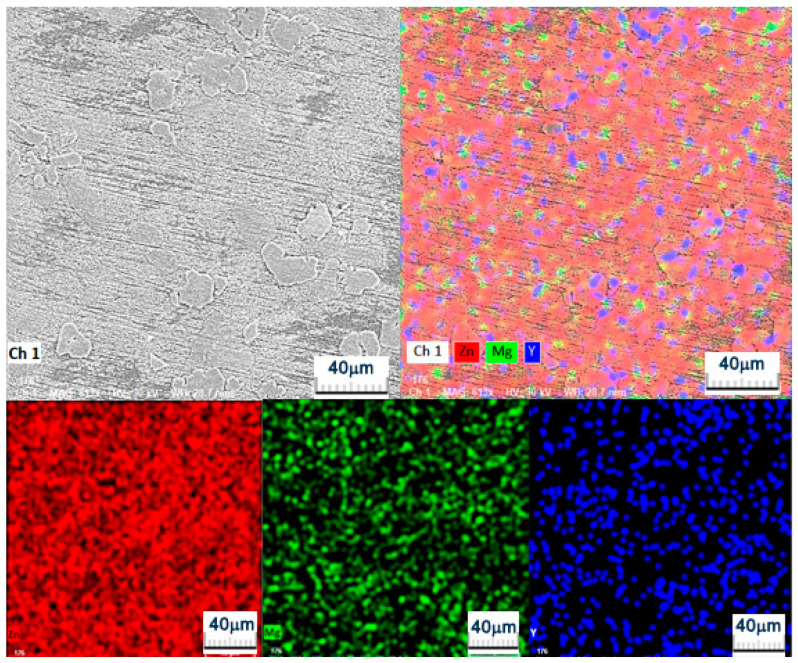
SEM microstructure of the Zn_3_Mg_0_._5_Y alloy showing dispersed Y-rich particles within the α-Zn matrix together with the corresponding elemental distribution obtained by EDS analysis.

Concurrently, the MgY master alloy used as an alloying addition may initially contain Mg–Y intermetallic compounds such as MgY, Mg_2_Y or Mg-rich phases like Mg_24_Y_5_. However, during the remelting process in the Zn-rich melt these particles are expected to dissolve due to the relatively low Mg content of the final alloy [25]. Consequently, under the magnesium-deficient conditions of the investigated Zn–3Mg–0.5Y composition, the persistence or formation of Mg–Y intermetallic particles in the final microstructure is considered unlikely, while the alloying elements are more likely to participate in the formation of Zn–Mg and Zn–Y intermetallic phases. Based on the EDS compositional analysis and the corresponding ZnMg and ZnY phase equilibria reported in the literature, the microstructure of the investigated Zn3Mg0.5Y alloy can be interpreted as consisting of a dendritic α-Zn matrix together with eutectic Zn–Mg intermetallic constituents (Mg_2_Zn_11_ and/or MgZn_2_) and dispersed Y-rich particles, most likely ZnY phases, while minor Mg–Y compounds cannot be completely excluded. The presence of these Y-rich phases is illustrated in Figure 3, where dispersed particles associated with Zn–Y intermetallic compounds can be observed within the Zn matrix and are confirmed by the corresponding elemental distribution analysis.

From a technological perspective, the utilisation of an electric furnace heated to approximately 400 °C and subsequently superheated to around 450 °C suggests the predominance of a Zn melt (i.e., a temperature above the melting point of Zn). In such a scenario, Mg (which has a significantly higher melting point) gradually dissolves or reactivates, and upon solidification, Mg and Y segregate to the final solidified liquid. This phenomenon elucidates the occurrence of secondary interdendritic phases. In conclusion, the microstructure of the alloy under investigation was found to be composed of dendritic α-Zn + eutectic Zn–Mg (Mg_2_Zn_11_/MgZn_2_) constituents, with dispersed Zn–Y (and possibly local Mg–Y) particles. The distribution of these particles was found to be controlled by homogenisation in the melt and the cooling regime [20,24].

**Figure 4:**
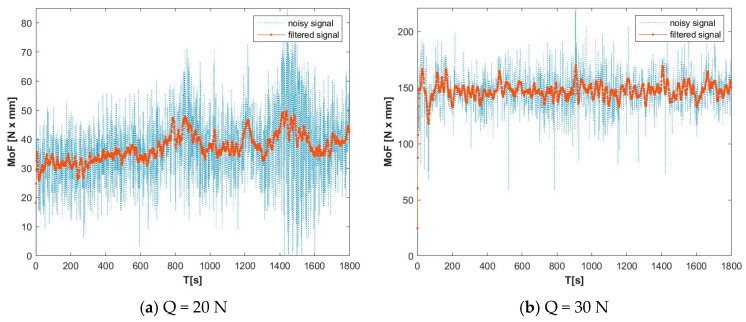
Filtered and raw (noisy) signals evolution for the moment of friction (MoF) of sample S09 at light load (**a**) and heavy load (**b**).

Figure 4 illustrates the variation in the unfiltered friction torque signal for an experimental sample at 20 N and 30 N. The observation indicates that, at an applied load of 20 N, the contact was initiated between the asperities of the mating surfaces. The friction process was found to be highly dynamic, resulting in a low CoF (Figure 4a) [26]. This result is validated by the obtained low wear rate of the sample at light load (20 N). As the load was increased to 30 N, the mating asperities were sheared, and the MoF signal became more stable, increasing in proportion to the load (Figure 4b).

It should be emphasized that the tribological tests performed in this study represent controlled dry sliding experiments intended primarily as a comparative screening of the intrinsic wear behaviour of the investigated alloys. While such tests provide valuable insight into the influence of alloy composition and microstructure on friction stability and material loss, they do not fully reproduce the complex conditions encountered in vivo. In physiological environments, additional factors such as corrosion processes, lubrication by biological fluids, cyclic loading and implant geometry can significantly modify the wear mechanisms. Therefore, the present results should be interpreted as an initial evaluation of tribological performance, which must be complemented by future tribocorrosion and physiological environment studies before drawing conclusions regarding clinical applications.

**Figure 5:**
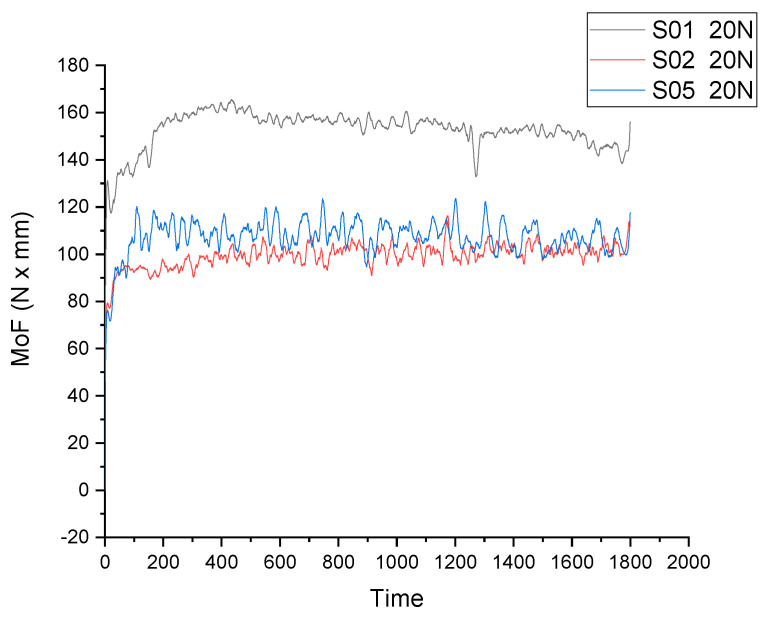
Moment of friction versus time for S01 (Zn pure), S02 (Zn3Mg) and S05 (Alloy III Zn3Mg0.5Y) at Q = 20 N.

**Figure 6:**
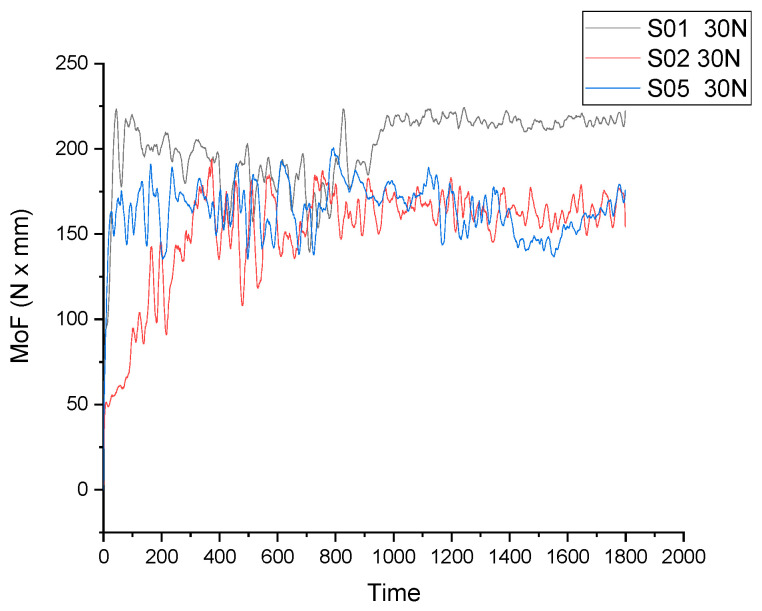
Moment of friction versus time for S01 (pure Zn), S02 (Zn3Mg) and S05 (Zn3Mg0.5Y) at Q = 30 N.

A comparison of the moment of friction variation over time for S01 (pure Zn), S02 (Zn3Mg) and S05 (Zn3Mg0.5Y) is presented for both loads in Figure 5 (Q = 20 N) and Figure 6 (Q = 30 N). In all cases, MoF (t) curves characteristically exhibit an initial run-in period (a rapid increase/adjustment) that is followed by a steady state period (oscillations around an average value). The disparities among materials become evident in two primary aspects: the mean MoF level, which is indicative of reduced friction; and the stability, signifying reduced oscillations indicative of enhanced contact with a more robust tribological layer. Additionally, the load sensitivity exhibits variation between 20 N and 30 N.

In comparison with pure Zn, adding Mg to Zn (Zn3Mg) results in a decrease in wear, as evidenced by the tribological behaviour of Zn–Mg alloys. At a load of Q = 30 N, the wear rate declines from 7.193 × 10^−4^ (pure cast Zn, S01) to 3.237 × 10^−4^ (cast Zn3Mg, S02), as demonstrated in Table 2. This enhancement is consistent with a more robust microstructure, characterised by the hardening of the Zn matrix through the formation of Zn-Mg-based intermetallic compounds. This process restricts local plastic deformation and material tearing at the point of contact [27,28].

In graphs, this is typically manifested as a more regulated MoF variation and a propensity towards a more stable state in comparison to pure Zn. Zn, being less rigid, can exhibit more significant variations in regime, contingent on material transfer and wear layer formation/degradation.

A similar outcome was observed in the presence of Mg, with the addition of Y resulting in a stable behaviour and low MoF for both loads (20 N and 30 N). In terms of wear (Q = 30 N), S05 exhibits a medium wear rate among the cast variants, significantly below pure Zn (S01) and slightly larger than Zn3Mg (S02). In such systems, tribological interpretation is frequently associated with microstructure stabilisation and/or the formation of constituents that can render the surface more resistant from a tribological point of view. This process simultaneously explains lower MoF (easier sliding/more uniform transfer), more stable MoF (reduced oscillations) and less wear (less mass loss) [29].

As demonstrated by the data presented in Figure 4 and Figure 5, it can be concluded that the S05 (Alloy III Zn3Mg0.5Y) sample exhibited a stable and lower friction coefficient for both 20 N and 30 N applied loads.

The observations recorded for Zn3Mg0.5Y (S05) indicate that the behaviour remains stable and exhibits low MoF at both loads (20 and 30 N). The comparison between cast and rolled Zn3Mg0.5Y demonstrates that the difference becomes pronounced at 30 N (lower MoF for rolled), while at 20 N they are similar. The comparative interpretation suggests that a higher load (30 N) results in more effective separation of materials based on the robustness of the tribological surface layer. Materials and conditions that can withstand contact without instability (particularly Zn3Mg0.5Y, and notably the hot rolled sample in terms of MoF) exhibit an advantage in terms of friction [30].

**Figure 7:**
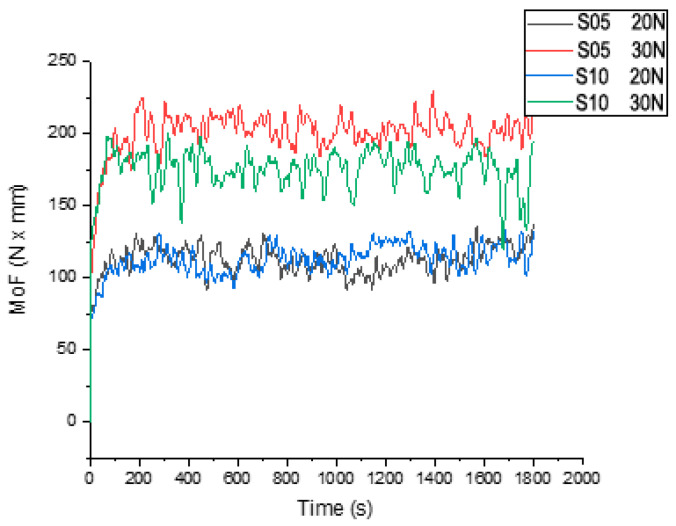
MoF values versus time for samples S05 (Zn3Mg0.5Y cast) and S10 (Zn3Mg0.5Y hot rolled) at Q= 20 N and Q = 30 N.

Finally, a comparison between the cast (S05) and hot rolled (S10) samples of Zn3Mg0.5Y is provided in Figure 7. The behaviour of both samples at a light load of 20 N is similar, but the hot rolled sample has a lower moment of friction at a heavier load (30 N) than the cast sample of Zn3Mg0.5Y.

As demonstrated in the comparison presented for Zn3MgY, at 20 N, the behaviour of the cast (S05) and rolled (S10) samples is described as being similar. At 30 N, the rolled sample exhibited a lower MoF compared to the cast sample (for the same type of Zn3Mg0.5Y alloy). This finding indicates that rolling becomes a particularly advantageous process when contact stress increases to 30 N, a phenomenon that is likely attributable to the compaction and homogenization of the surface layer, the enhancement of resistance to local plastic deformation, and the stabilisation of contact, as evidenced by a reduction in stick–slip events. In practice, a reduction in friction does not necessarily result in a corresponding reduction in wear; this is contingent on the specific mechanism in operation (e.g., adhesion, abrasion, film stability, local embrittlement, etc.) [31].

The samples were weighted prior to and following the friction test in order to calculate the wear rate. For a constant speed of 100 rpm, the total sliding distance in each friction test was 556 metres. The results for a constant load Q = 30 N are presented in Table 2. The wear rate was expressed using the well-known Archard’s law (Equation (1)):

(1) $$W=\frac{M}{Q\cdot D},$$

where W is the wear rate, M represents the mass loss during one test, in grams, Q is the applied normal load in Newtons, and D is the sliding distance (556 m).

In implant–bone or implant–implant contacts, two factors are of particular significance: low wear (fewer particles/debris, less material loss) and a more stable MoF variation over time (more predictable contact, fewer episodes of stick–slip that can accelerate wear and induce micro-movements). As demonstrated in the findings reported at Q = 30 N, the cast Zn3Mg (S02) sample exhibited the lowest wear rate among the variants examined, with a value of 3.237 × 10^−4^. This sample was characterised by its minimal MoF and stability under both loads of 20 and 30 N. The cast Zn3Mg0.5Y alloy (S05) exhibited intermediate wear (5.755 × 10^−4^), while the cast pure Zn (S01) demonstrated higher wear (7.193 × 10^−4^). For the rolled Zn3Mg0.5Y (S10) sample, the MoF is observed to be lower at 30 N in comparison to the cast sample. However, the wear rate at 30 N is found to be higher than that of the S05 sample (5.035 × 10^−4^ vs. 3.237 × 10^−4^).

The differences observed in the wear behaviour of the investigated alloys can be correlated with the microstructural features generated by alloying. Pure Zn exhibits relatively low hardness and a predominantly single-phase α-Zn structure, which promotes plastic deformation and adhesive wear during sliding contact. The addition of Mg leads to the formation of Zn–Mg intermetallic compounds (such as Mg_2_Zn_11_ or MgZn_2_), which strengthen the matrix and reduce the extent of plastic deformation at the contact interface. As a result, the Zn–3Mg alloy shows improved wear resistance compared to pure Zn. The further addition of Y contributes to microstructural stabilization through the formation of dispersed Y-rich particles and possible grain refinement effects. Although these phases may not always significantly reduce the total wear volume, they can increase the stability of the friction response and influence the formation of the tribological surface layer. In addition, thermomechanical processing such as hot rolling can further modify the microstructure by improving phase distribution and reducing structural heterogeneity, which explains the more stable friction behaviour observed for the rolled Zn_3_Mg_0.5_Y alloy.

**Figure 8:**
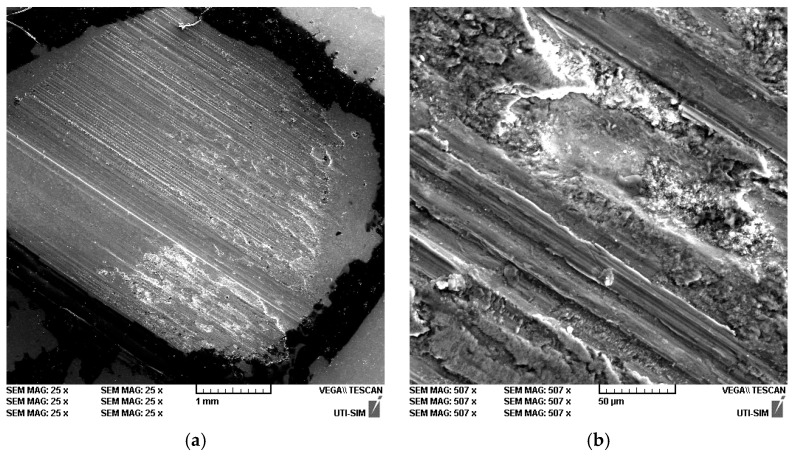
SEM images of the wear mark (**a**) 25×—general view and (**b**) 500×—detail of the mark.

The mark exhibits a rectangular strip shape with clear definition, accompanied by parallel striations that extend across the majority of its width, thereby delineating the direction of sliding. This phenomenon is indicative of abrasion wear, specifically the micro-ploughing effect. There are shinier/lighter areas inside the mark, especially towards the bottom and in the central area. As illustrated in Figure 8a, a few micro-cavities emerge at the right edge, indicating either local tearing or the detachment of particles (pull-out) during friction [32]. In conclusion, the trace is dominated by directional abrasion, but with clear indications of local recompacting and point-like defects (debris/pull-out), so it is not perfectly uniform pure abrasion [33]. When the image of the track is subjected to enlargement (Figure 8b), the mechanism becomes more pronounced: deep, continuous grooves (micro-cutting/micro-ploughing) become visible, and “raised” material appears on the flanks, thereby confirming the presence of an abrasive component. In the central area, there is a region that exhibits a granular, uneven appearance, accompanied by recompacted debris and irregular edges. This is indicative of a tribo-layer (tribological layer), which is characterised by the accumulation of pressed wear particles in a localised manner [34]. Additionally, there are areas of extensive and smeared surface, indicating instances of adhesion and transfer (micro-scale metal-to-metal contact) which were subsequently affected by abrasion. It has been confirmed that a mixed wear mechanism is in operation, whereby abrasion produces grooves and adhesion and recompacting generate tribo-layer/debris areas. This is, in practice, a classic scenario for softer alloys in dry contact, involving abrasion and the formation and subsequent rupture of a local oxide/debris layer.

As demonstrated in Figure 8, the wear observed during the test was measured between the Zn3Mg0.5Y alloy and the AISI 52100 roller (62–65 HRC) at 20 N. In such contact, wear occurs predominantly on the softer sample (Zn3Mg0.5Y). The 52100 steel disc (which possesses a high degree of hardness and is of the bearing type) exhibits minimal wear, yet it has the capacity to perform the following functions: cut/groove the Zn3Mg0.5Y surface, promote material transfer (Zn and oxides) onto the disc, and generate fine debris that subsequently acts as third-body abrasion. At 20 N, the load is moderate but sufficient to cause initial running-in, oxide film formation, and stabilization [35]. The parallel striations located at the rear exhibit seamless congruence with the grooves produced by the inherent roughness of the 52100 steel and/or the presence of hard particles that have been brought into contact. The raised edges of the grooves indicate ploughing (deformation and lateral pushing) rather than clean removal, such as chipping. This is typical when a soft material slides over a very hard one [36]. The presence of granular nonuniform areas and small cavities can be attributed to the formation and compaction of oxide debris, as well as the local tearing of more rigid constituents from the microstructure, particularly in regions containing Y-rich phases.

**Figure 9:**
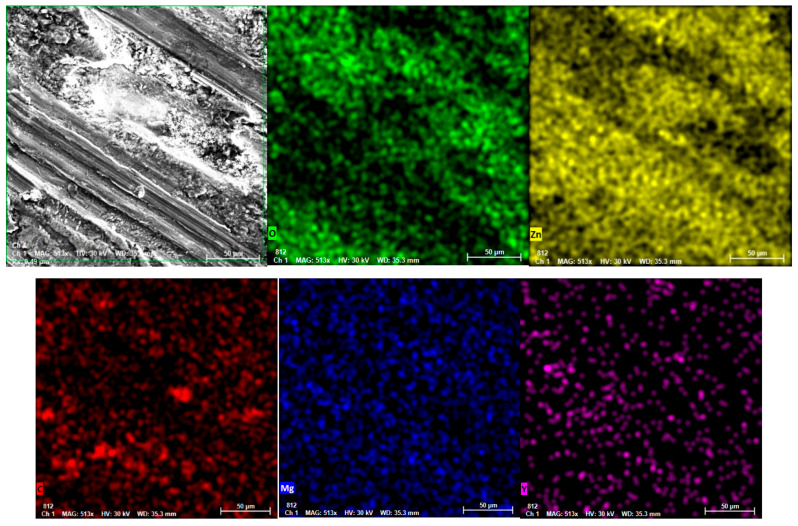
Elemental distribution of the elements on the wear mark.

Figure 9 illustrates the distribution of elements following wear, demonstrating that the oxygen signal is dispersed across the entire analysed area, exhibiting variations in intensity. This indicates the presence of an oxide/tribo-oxide film on the track, resulting from oxidation during friction and recompaction. This layer is typically advantageous in terms of stabilization, with its continuity leading to a reduction in adhesion. Conversely, its fracturing during impact results in the formation of debris. Zinc has been found to be present in a uniform manner throughout the matrix, with no significant variations observed. Such uniformity can be attributed to the fact that the analysed area is predominantly composed of the Zn-α matrix, which does not constitute a substantial foreign deposit. Instead, it is a composite of matrix, oxides, and formed debris. Magnesium exhibits a fine, relatively homogeneous distribution across the EDS field. Such distribution is attributable to Mg’s presence in a single area, as well as its dispersion throughout the trace area. This is consistent with a contribution to the tribological film/local oxides. The distribution of yttrium compounds is found to be uneven due to the association of Y with Y-rich constituents (phases/intermetallics/precipitates), which exhibit higher hardness compared to the Zn matrix and demonstrate enhanced stability within the worn area [37,38]. From a tribological perspective, these regions are prone to functioning as harder micro-bearing areas, thereby assisting in the mitigation of local plastic deformation. Carbon exhibits unevenness, with more pronounced regions suggesting the presence of local deposits rather than a homogeneous carbon layer [39,40,41].

**Figure 10:**
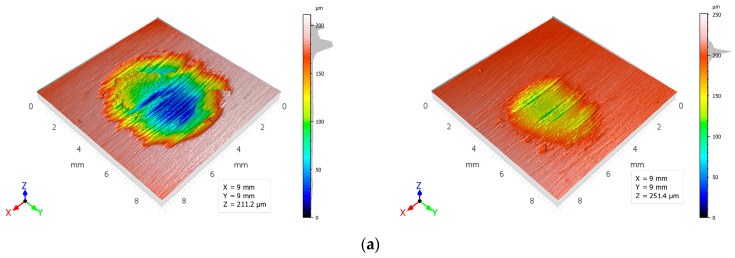

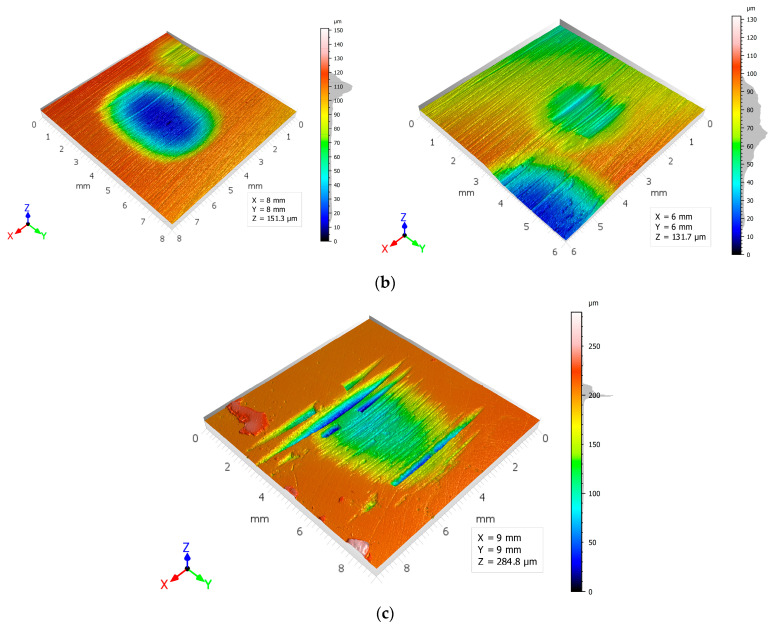
3D profiles of the wear mark (**a**) pure Zn, (**b**) Zn3Mg alloy and (**c**) Zn3Mg0.5Y alloy.

Furthermore, pure Zn demonstrated heightened sensitivity to load augmentation (from 20 to 30 N), exhibiting a discernible alteration in roughness value (Ra) consequent to plastic deformation and adhesive wear, as illustrated in Figure 10a and substantiated in Table 3. The Zn–3Mg material exhibited more stable Ra values on the wear mark between the two load conditions of 20 and 30 N, suggesting more controlled tribological behaviour and better resistance to topography degradation. At 30 N, the order of wear trace roughness values is as follows: The Zn–3Mg composition exhibits a lower concentration of Zn than that observed in pure Zn, which itself is significantly lower than that of Zn–3Mg–0.5Y. The elevated roughness of Zn–3Mg–0.5Y suggests the presence of more aggressive wear, likely dominated by abrasive mechanisms and/or delamination associated with its complex microstructure [42].

**Figure 11:**
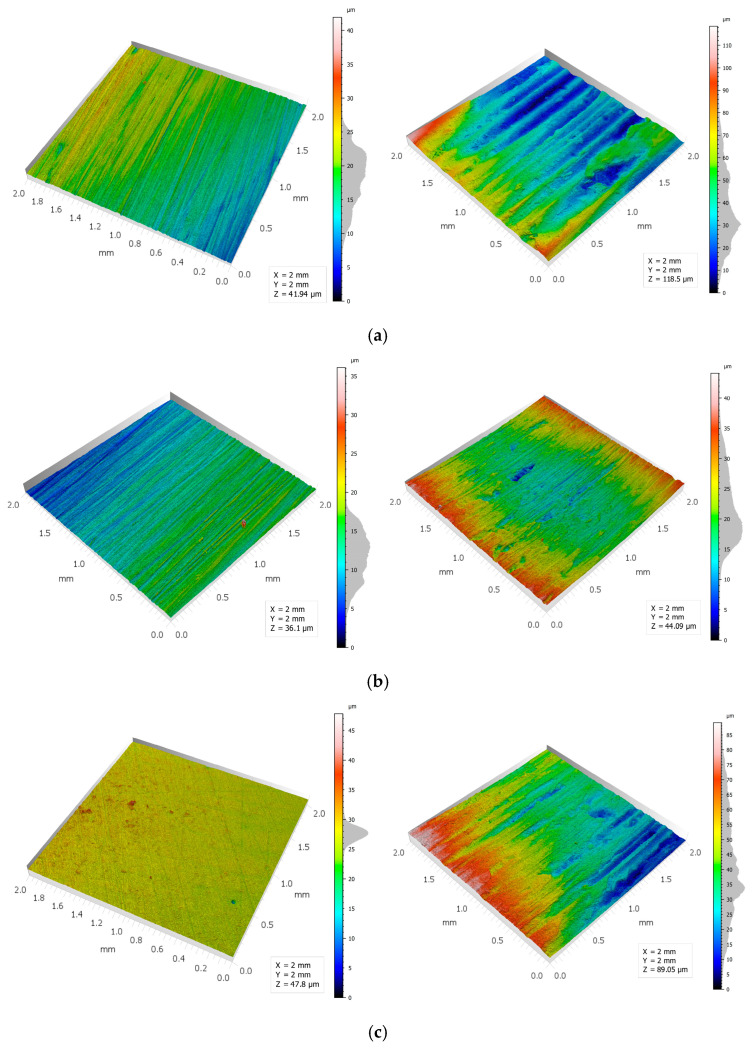
3D profiles of the surfaces of (**a**) pure Zn, (**b**) Zn3Mg, and (**c**) Zn3Mg0.5Y for the unused surface on the left and the used surface on the right.

The load effect for the same material is illustrated in Figure 11. In the case of pure Zn, the volume lost increases from 0.533 mm^3^ (20 N) to 2.282 mm^3^ (30 N), i.e., approximately 4.3×, indicating a pronounced sensitivity of pure Zn to increased contact (plastic deformation + more severe material removal at 30 N). As the Zn3Mg alloy is subjected to different levels of force, the volume lost increases from 0.0907 mm^3^ (20 N) to 0.967 mm^3^ (30 N). This increase is approximately 10.7 times greater than that of pure zinc. This is due to the more pronounced hardening of the Zn3Mg alloy after hot-rolling in comparison to that of pure zinc.

A consistency check between the mass loss measurements and the wear volume obtained by optical profilometry was performed using the density of zinc-based alloys (≈7.1 g·cm^−3^). For example, for pure Zn tested at 30 N the measured mass loss of 0.020 g corresponds to an estimated wear volume of approximately 2.8 mm^3^ (Δm/ρ), which is in reasonable agreement with the profilometrically measured value of 2.28 mm^3^. The small difference between the two approaches can be attributed to local surface irregularities, boundary definition of the wear scar and the presence of compacted debris within the wear track.

**Figure 12:**
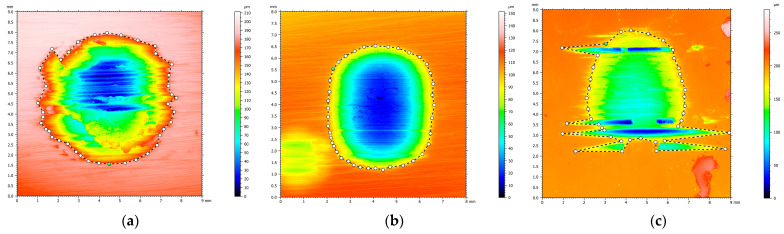
Loss volume determination for (**a**) pure Zn, (**b**) Zn3Mg and (**c**) Zn3Mg0.5Y.

At 20 N, the alloy functions in a markedly moderate manner (exhibiting an extremely low loss); however, at 30 N, it transitions into a considerably more severe mode (presumably marking the transition threshold to more intense wear). The comparison of materials at equivalent loads of 20 N reveals that the Zn–3Mg alloy (0.091 mm^3^) exhibits a loss that is approximately 5.9 times smaller than that observed in pure Zn (0.533 mm^3^). The result is consistent with the hardened microstructure and higher hardness of Zn–Mg, which limits volume removal under moderate conditions. In the case of more pronounced wear, for example at 30 N, the order of volume loss at a load of 30 N is Zn–3Mg < Zn–3Mg–0.5Y < pure Zn, thus demonstrating that Zn–3Mg is the most effective in limiting volume loss under high load. This supports the role of Mg in hardening and stabilising wear behaviour. The Zn-3Mg-0.5Y alloy exhibited a reduced volume loss relative to pure Zn, though slightly higher than Zn-3Mg, indicating that the incorporation of Y does not ensure a guaranteed reduction in volume loss under severe conditions. The presence of a more intricate microstructure, characterised by hard phases and heterogeneities, may promote local fragmentation and third-body wear, thereby maintaining the volume loss at an intermediate level. The volume loss was determined through the utilisation of the optical profilometer software, v. 5.7.8.31265, as depicted in Figure 12.

The variation in the R_q_ parameter (RMS roughness) on the wear marks highlights both the effect of the load and the influence of the microstructure of each material. For the pure Zn sample, an increase in force from 20 N to 30 N results in an enhancement of Rq from approximately 5.41 µm to 7.04 µm, signifying an intensification of the high-amplitude components of the topography (deeper grooves and more irregular relief) under more severe contact [43]. The R_q_ value of Zn–3Mg is significantly lower than that of pure Zn for both loads, exhibiting a moderate increase from approximately 2.19 µm (20 N) to approximately 2.06 µm (30 N). This range remains practically constant, indicating a more regulated wear process and a more stable surface. This is compatible with the hardening of the Zn matrix by ZnMg compounds and the reduction in plastic deformation. A comparison of the materials at 30 N, from the perspective of R_q_ values, reveals that the Zn–3Mg–0.5Y specimen exhibits a notably elevated R_q_ value, indicative of a wear trace characterised by substantial variations in height. Such variations are associated with more pronounced wear, as evidenced by the presence of significant abrasion and fragmentation of constituents, as well as the manifestation of the third-body effect. These phenomena serve to amplify the mean square deviations of the profile.

The R_sk_ (skewness) parameter, as outlined in Table 3, quantifies the degree of asymmetry in the distribution of roughness profile heights relative to the mean line. R_sk_ values greater than 0 indicate a surface dominated by peaks (more protrusions than average), while R_sk_ values less than 0 indicate a surface dominated by valleys/trenches (more pronounced depressions below the average). In all wear marks, the analysed materials demonstrate a tendency to exhibit negative values. This finding indicates that wear predominantly generates microchannels and depressions (i.e., ploughing/micro-cutting) rather than peaks. For pure Zn, R_sk_ becomes more negative as the load increases from 20 N (≈−1.58) to 30 N (≈−0.80), suggesting that for lower stresses there is a higher proportion of deep valleys relative to the average level, while at 30 N, plastic deformation and smearing contribute more, which can partially fill the depressions and make the distribution less asymmetrically negative. For Zn–3Mg, the transition from 20 N (≈−0.93) to 30 N (≈−0.33) shows the same trend: at higher loads, the profile becomes closer to a symmetrical distribution, consistent with a more uniform wear pattern and better resistance to deep groove formation. Comparing the coefficient values for the 30 N load, Zn–3Mg has the least negative R_sk_ (≈−0.33), indicating a relatively more balanced trace, while pure Zn has a more negative R_sk_ (≈−0.80), thus a topography more dominated by depressions. For Zn–3Mg–0.5Y, the R_sk_ value is higher (≈−2.88), suggesting a wear pattern strongly dominated by deep valleys/grooves, consistent with a more severe wear regime (marked abrasion and/or local detachments), which shifts the height distribution towards below-average values [44].

The R_ku_ parameter (kurtosis) describing the sharpness of the distribution of roughness profile heights is an indicator of the presence of extreme elements. For an ideal Gaussian distribution, R_ku_ ≈ 3; R_ku_ values > 3 indicate a surface with rare but very pronounced events (high peaks or deep valleys), while values close to 3 indicate a more statistically uniform topography. In terms of wear marks, all materials generally exhibit an R_ku_ significantly greater than 3, confirming that wear produces a surface dominated by local discontinuities (deep micro-grooves, tears, adhering fragments, or deposits). For pure Zn, the R_ku_ coefficient varies from approximately 13.32 (20 N) to 9.22 (30 N), suggesting that at 20 N the topography includes more local extremes (isolated depressions/peaks), while at 30 N plastic deformation and material transfer can partially smooth out the profile, reducing kurtosis. In the case of Zn–3Mg, R_ku_ is more moderate and varies from ~5.91 (20 N) to ~8.76 (30 N), indicating that at higher loads more extreme characteristics associated with increased abrasion and micro-fragmentation occur, even though the overall amplitude of roughness remains relatively low compared to pure Zn. At 30 N, the differences between materials are evident: Zn–3Mg has a lower R_ku_ than pure Zn, suggesting a wear trace less dominated by extreme defects, while for Zn–3Mg–0.5Y, the R_ku_ remains high (≈12.40), confirming a surface with numerous severe topographical events (deep valleys and abrupt variations), consistent with a more aggressive wear regime and a more heterogeneous microstructure.

In the context of application types involving implant–bone, such as temporary implants in contact with bone (e.g., screws, pins, thin plates, fasteners), the objective of achieving friction stability and a reduced wear process (i.e., a reduction in the number of particles) is of paramount importance. In this regard, Zn–3Mg demonstrates the lowest wear rate, while Zn–3Mg–0.5Y exhibits enhanced friction stability with intermediate wear. Pure Zn exhibited higher wear and less stable friction behaviour under the present dry sliding conditions. These results suggest that alloying with Mg may be beneficial for improving tribological performance. However, the suitability of pure Zn or Zn-based alloys for specific biomedical applications must be evaluated through additional studies under simulated physiological conditions. The Zn3Mg0.5Y alloy showed relatively stable friction behaviour and moderate wear under the investigated dry sliding conditions. These characteristics may indicate a favourable tribological response, although further studies under physiological environments are required before considering potential biomedical applications.

A thorough examination of the outcomes derived from numerous recommendations concerning interactions with ceramic materials, stainless steel, and Ti-based alloys has led to the adoption of a cautious approach. This approach acknowledges the significance of the tribological pair, often being as crucial as the implant material itself. This is due to the fact that substantial disparities in hardness can result in alterations to the wear mechanism, encompassing processes such as abrasion, adhesion, and transfer. In the event of zinc-based elements coming into contact with ceramic materials (e.g., hydroxyapatite, alumina, or zirconia), which are generally much harder than Zn-based alloys, the classic risk is that the metal (Zn alloy) will be the part that wears out. These observations suggest that the tribological behaviour of Zn-based alloys may strongly depend on the counterface material and hardness mismatch. However, the present experiments were performed only against AISI 52100 bearing steel under dry sliding conditions. Therefore, the interaction of these alloys with ceramics, titanium or stainless steel surfaces should be considered hypothetical and requires dedicated tribological investigations under application-specific conditions. When considering medical applications involving repeated contact or significant sliding, it is advisable to either avoid direct contact (by design) or to consider an intermediate surface or treatment (low roughness, possibly a protective coating), as ceramics have been shown to induce wear through abrasion. In the hypothetical case of a medical application where the alloy comes into contact with stainless steel, the difference in hardness is again to the disadvantage of the Zn alloy, especially at higher loads (30 N). Titanium is known to cause galling and adhesion when in contact with certain metal–metal pairs. In practice, difficult metal–metal pairs are avoided and surface films are stabilized. In the case of potential contact with Ti-based components, differences in hardness and adhesion behaviour could influence the wear mechanism. However, the present study does not include such tribological pairs, and therefore further experimental studies would be required to evaluate the behaviour of Zn-based alloys in these conditions.

The mass loss, wear rate, and wear volume values reported for each condition correspond to individual tested specimens, whereas future work will include at least three independent replicates per condition in order to provide a full statistical analysis of tribological performance.

## 4. Conclusions

The dry sliding wear behaviour of pure Zn, Zn–3Mg, and Zn–3Mg–0.5Y alloys was systematically evaluated in order to assess the influence of alloying and processing on friction stability and material loss under controlled contact conditions. The outcomes of this study demonstrate that alloying Zn with Mg significantly improves wear resistance in comparison with pure Zn. This is primarily due to matrix strengthening by Zn–Mg intermetallic phases, leading to reduced plastic deformation and more stable surface topography. While the addition of Y has been shown to contribute to a more stable friction response and reduced fluctuations of the friction torque, it does not consistently result in lower wear volume under higher load. In such cases, the more complex and heterogeneous microstructure promotes localized fragmentation and third-body abrasion.

Zn–3Mg was found to exhibit the lowest wear rate at 30 N, while Zn–3Mg–0.5Y demonstrated an intermediate wear rate coupled with enhanced friction stability. Surface and subsurface analyses were conducted to ascertain the wear mechanism, which was found to be a combination of abrasion, oxide tribolayer formation, and localized adhesion. The findings indicate that Mg plays a primary role in enhancing wear resistance, whereas Y mainly contributes to friction stabilisation rather than further reducing material loss. The results obtained in this study provide a reliable baseline for the tribological performance of Zn-based biodegradable alloys. Furthermore, they support the need for future studies to be conducted under combined wear and corrosion conditions for the purpose of more accurately simulating in vivo environments.

From a materials design perspective, the present results indicate that the wear resistance of Zn-based biodegradable alloys can be improved through controlled alloying and microstructural engineering. The addition of Mg plays a key role by promoting the formation of Zn–Mg intermetallic phases, which strengthen the α-Zn matrix and reduce plastic deformation during sliding contact. Minor additions of rare earth elements such as Y may further contribute to microstructural stabilization through the formation of dispersed Y-rich particles and by influencing the stability of the tribological surface layer. Consequently, the combination of Mg alloying and controlled additions of rare earth elements represents a promising strategy for improving the tribological performance of biodegradable Zn alloys intended for biomedical applications.

## Figures and Tables

**Table 1:** The chemical composition of the alloy.

Area/Elements	Zn			Mg			Y		
	wt%	at%	Err.	wt%	at%	Err.	wt%	at%	Err.
Area 1	97.032	93.43	2.30	2.38	6.15	0.22	0.59	0.42	0.26
Area 2	96.96	93.48	2.23	2.31	6.00	0.22	0.73	0.52	0.261
Area 3	96.66	91.51	2.27	3.33	8.48	0.28	0.01	0.01	0.11
Area 4	96.45	93.11	2.15	2.32	6.01	0.21	1.23	0.87	0.27
Area 5	97.07	93.31	2.32	2.46	6.36	0.23	0.47	0.33	0.26
Average (SD)	96.84 (±0.27)	92.968 (±0.83)	2.255 (±0.07)	2.558 (±0.44)	6.601 (±1.06)	0.233 (±0.03)	0.607 (±0.44)	0.430 (±0.31)	0.232 (±0.07)

**Table 2:** Mass loss and wear rate for various tested samples at Q = 30 N.

Sample	Notation	Mass, Grams		Mass Loss, Grams, M	Relative Mass Loss, wt. %	Wear Rate, $W=\frac{M}{Q\cdot D}$
		Initial	Final			
Zn (pure) cast state	S01	18.373	18.353	0.020 ± 0.001	0.1089	7.193 × 10^−4^
Zn3Mg cast state	S02	12.470	12.461	0.009 ± 0.001	0.0722	3.237 × 10^−4^
Zn3Mg0.5Y cast state	S05	7.270	7.254	0.016 ± 0.001	0.2201	5.755 × 10^−4^
Zn (pure, hot rolled)	S08	7.520	7.498	0.022 ± 0.001	0.2926	7.913 × 10^−4^
Zn3Mg hot rolled	S09	11.964	11.952	0.012 ± 0.001	0.1003	4.316 × 10^−4^
Zn3Mg0.5Y hot rolled	S10	15.759	15.745	0.014 ± 0.001	0.0888	5.035 × 10^−4^

Note: Mass measurements were performed using an analytical balance with a precision of ±0.001 g.

**Table 3:** Characteristics of the surfaces of the experimental materials (before and after wear) (Three determinations were made for the surface texture parameters (Ra, Rq, Rsk and Rku), and standard deviation is reported for these quantities. Lost volume values correspond to the analysed wear track for each tested specimen).

Material	Force (N) Condition/ Analyzed Area	R_a_ (µm)	R_q_ (µm)	R_sk_ (-)	R_ku_ (-)	Lost Volume (mm^3^)
Zn (pure)	Initial surface	0.92 ± 0.18	1.23 ± 0.23	−0.15 ± 0.06	5.73 ± 1.19	-
Zn (pure)	30 N/Wear mark	4.47 ± 1.78	7.04± 0.57	−0.80 ± 0.71	9.22 ± 3.10	2.28
Zn (pure)	Initial surface	0.91 ± 0.15	1.3 ± 0.16	−1.04 ± 0.56	9.07 ± 5.68	-
Zn (pure)	20 N/Wear mark	3.56 ± 1.5	5.41 ± 0.52	−1.58 ± 0.27	13.32 ± 1.71	0.53
Zn–3Mg	Initial surface	0.91 ± 0.21	1.23 ± 0.22	−0.78 ± 0.61	5.67 ± 1.93	-
Zn–3Mg	30 N/Wear mark	1.30 ± 0.36	2.06 ± 0.51	−0.33 ± 0.01	8.76 ± 1.13	0.97
Zn–3Mg	Initial surface	1.09 ± 0.27	1.42 ± 0.29	−0.34 ± 0.64	4.12 ± 0.47	-
Zn–3Mg	20 N/Wear mark	1.41 ± 0.67	2.19 ± 1.03	−0.93 ± 0.76	5.91 ± 0.81	0.09
Zn–3Mg–0.5Y	Initial surface	0.6 ± 0.053	0.89 ± 0.2	−1.27 ± 0.76	14.81 ± 2.05	-
Zn–3Mg–0.5Y	30 N/Wear mark	10.25 ± 1.74	18.96 ± 9.01	−2.88 ± 0.80	12.4 ± 2.75	1.17

## Data Availability

The original contributions presented in this study are included in the article. Further inquiries can be directed to the corresponding authors.
